# Chain Entanglement of 2-Ethylhexyl Hydrogen-2-Ethylhexylphosphonate into Methacrylate-Grafted Nonwoven Fabrics for Applications in Separation and Recovery of Dy (III) and Nd (III) from Aqueous Solution

**DOI:** 10.3390/polym12112656

**Published:** 2020-11-11

**Authors:** Hiroyuki Hoshina, Jinhua Chen, Haruyo Amada, Noriaki Seko

**Affiliations:** Department of Advanced Functional Materials Research, Takasaki Advanced Radiation Research Institute, Quantum Beam Science Research Directorate, National Institutes for Quantum and Radiological Science and Technology, 1233 Watanuki-machi, Takasaki, Gunma 370-1292, Japan; amada.haruyo@qst.go.jp (H.A.); seko.noriaki@qst.go.jp (N.S.)

**Keywords:** selective adsorption, dysprosium, neodymium, fabric adsorbent, radiation, graft polymerization

## Abstract

A nonwoven fabric adsorbent loaded with 2-ethylhexyl hydrogen-2-ethylhexylphosphonate (EHEP) was developed for the separation and recovery of dysprosium (Dy) and neodymium (Nd) from an aqueous solution. The adsorbent was prepared by the radiation-induced graft polymerization of a methacrylate monomer with a long alkyl chain onto a nonwoven fabric and the subsequent loading of EHEP by hydrophobic interaction and chain entanglement between the alkyl chains. The adsorbent was evaluated by batch and column tests with a Dy (III) and Nd (III) aqueous solution. In the batch tests, the adsorbent showed high Dy (III) adsorptivity close to 25.0 mg/g but low Nd (III) adsorptivity below 1.0 mg/g, indicating that the adsorbent had high selective adsorption. In particular, the octadecyl methacrylate (OMA)-adsorbent showed adsorption stability in repeated tests. In the column tests, the OMA-adsorbent was also stable and showed high Dy (III) adsorptivity and high selectivity in repeated adsorption–elution circle tests. This result suggested that the OMA-adsorbent may be a promising adsorbent for the separation and recovery of Dy (III) and Nd (III) ions.

## 1. Introduction

Rare earths including scandium, yttrium, and 15 lanthanoid elements, have recently become indispensable materials for the high-tech industry. Due to the uneven distribution of rare-earth sources in the world, almost all rare earths are supplied by limited countries [1]. Therefore, it is necessary to recycle used rare earths to ensure a stable supply of these materials in many countries [2,3,4,5,6,7]. Among the rare earths, dysprosium (Dy) and neodymium (Nd) are listed as “critical materials” by the United States due to supply issues and their importance to electronics and electrical technology [8,9]. For example, neodymium and dysprosium are key components of permanent magnets, such as NdFeB magnets. The demand for the separation and recovery of dysprosium and neodymium from used permanent magnets and scraps generated during manufacturing is increasing [10,11,12,13,14,15,16,17,18,19,20].

The technology for the separation and recovery of dysprosium and neodymium from used permanent magnets has been extensively studied [21,22]. The most common method of recovering dysprosium and neodymium from waste materials involves leaching them in an acid solution and purifying the leached ions by solvent extraction [11,12,13,23]. Organophosphorus compounds such as 2-ethylhexyl hydrogen-2-ethylhexylphosphonate and di(2-ethylhexyl)phosphoric acid, carboxylic acid such as neodecanoic acid and naphthenic acid, and methyltrioctylamine chloride are usually used as extractants for rare-earth ions due to their good separation and recovery performance [22,23,24,25,26]. However, solvent extraction requires a large number of separation steps, a long processing time, and a large space for all necessary equipment. On the other hand, other methods such as chemical precipitation and ionic liquids extraction are also used for the separation and recovery of rare earths. Although the chemical precipitation process is simple and low in cost, the purity and recovery ratio of the resulting product are usually low, while the ionic liquid extraction cost is high for actual application [21]. Currently, the effective separation and recovery of rare earths from an aqueous solution requires relatively simple processes [27,28]. Adsorption techniques using adsorbents, such as inorganic particles, ion-exchange resins, and polymer ligands, are attractive for the separation and recovery of rare-earth ions [29,30,31,32,33,34,35,36]. This is because the adsorption process does not require much energy and water and can be easily operated anywhere by batch or column methods [37].

Inorganic particles, such as clay minerals, activated carbon, and magnetite nanoparticles, are highly suitable for removing heavy metals from water and wastewater. In many cases, these inorganic materials show high adsorption but low selectivity [37,38,39]. On the other hand, adsorbents with special ligands or chelating functional groups can be designed to selectively separate and recover target metal ions in water. These adsorbents, including ion-exchange resins and polymer ligands, can be prepared by introducing functional groups onto polymer materials by the radiation-induced graft polymerization method. This method can introduce new functional properties while maintaining the properties of the trunk polymers [40,41,42,43,44,45,46,47,48]. Various vinyl monomers have been radiation-grafted onto trunk polymers, such as polyethylene [41,42], polypropylene [43,44], fluoropolymers [45], and cellulose [46,47]. Furthermore, graft polymerization can be applied to various types of materials, such as films [45], fabrics [30,45,46,47], fibers [46], and particles [48]. Various adsorbents have been developed using this technology for the recovery and removal of metal ions from environmental water and industrial wastewater [46,47,48,49,50,51]. In the design of these adsorbents, it is important to select the most suitable functional groups based on the metal ion that needs to be adsorbed. 

We noticed that 2-ethylhexyl hydrogen-2-ethylhexylphosphonate (EHEP), used as an extractant in the solvent extraction process, has two alkyl chains on each molecule [10,24,52]. In this study, we attempted to load EHEP onto polyethylene-coated polypropylene (PE/PP) nonwoven fabrics to develop a novel adsorbent for rare-earth ions. For this purpose, we grafted a polymerized methacrylate monomer with a long alkyl chain onto the fabrics. The EHEP was then loaded onto the grafted fabrics by hydrophobic interaction and chain entanglement between the alkyl chains. Here, since the EHEP is only physically bonded on the fabrics by hydrophobic interaction and chain entanglement, the loss of EHEP is a concern in practical applications. Therefore, the stability of EHEP-loaded adsorbents needs to be confirmed for practical use.

Four methacrylate monomers with different alkyl chain lengths—butyl methacrylate (BMA), hexyl methacrylate (HMA), dodecyl methacrylate (DMA), octadecyl methacrylate (OMA)—were radiation-grafted onto the PE/PP nonwoven fabrics in this study. The grafted fabrics were then loaded with EHEP to prepare the adsorbents. The adsorbents were tested in batch and column modes using Dy (III) and Nd (III) ion solutions [18]. The effects of the alkyl chain length of the monomers on the stability and adsorption performance of the EHEP-loaded absorbents were studied and evaluated.

## 2. Experimental

### 2.1. Materials

The trunk material used for graft polymerization was a nonwoven fabric composed of polyethylene-coated polypropylene (PE/PP) fibers, provided by Kurashiki Textile Manufacturing Co., Ltd., Kurashiki, Japan. The PE on the fiber surface is easy to be radiation-grafted, and the PP core makes the fiber mechanically stronger. Furthermore, the PE/PP nonwoven fabric is relatively cheap among artificial fabrics and has a large specific surface. The four methacrylate monomers—butyl methacrylate (BMA), hexyl methacrylate (HMA), dodecyl methacrylate (DMA), and octadecyl methacrylate (OMA)—are of chemical reagent grade and were purchased from Fujifilm Wako Pure Chemical Corporation, Tokyo, Japan. 2-Ethylhexyl hydrogen-2-ethylhexylphosphonate (EHEP) was provided by Daihachi Chemical Industry Co., Ltd., Tokyo, Japan. The other reagents, such as Tween 20 surfactant, methanol, ammonia water, HCl solution, Dy (III) (Dy_2_O_3_ in 5 wt.% HNO_3_) standard solution, and Nd (III) (Nd_2_O_3_ in 5 wt.% HNO_3_) solution, were purchased from Kanto Chemical Co., Inc., Tokyo, Japan. All chemicals were used without further purification. In this study, the deionized Mili-Q water with a high resistivity of 18 MΩ cm was used. 

### 2.2. Graft Polymerization of Methacrylate Monomers

**Figure 1:**
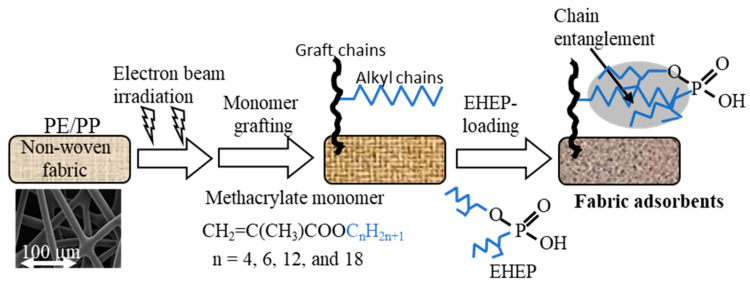
Preparation of the fabric adsorbents by graft polymerization of methacrylate monomers and the subsequent 2-ethylhexyl hydrogen-2-ethylhexylphosphonate (EHEP) loading.

Figure 1 shows the process of preparing the fabric adsorbents. Graft polymerization was performed using a preirradiation method. In this study, either PE nonwoven fabric or PP nonwoven fabric could be used as trunk polymers. However, the mechanical strength of common PE nonwoven fabric is significantly lower than that of PP nonwoven fabric, while the PP nonwoven fabric deteriorates faster than PE nonwoven fabric. Therefore, we chose the PE-coated PP nonwoven fabric as the polymer trunk for radiation grafting. The PE/PP nonwoven fabric with a size of 5 cm × 8 cm was placed in a polyethylene bag, purged with nitrogen gas to create an oxygen-free environment, and electron beam preirradiated at −80 °C (dry ice) with a beam energy of 2 MeV at a current of 3 mA to generate radicals on the fabric. The preirradiated fabric was removed and filled into a glass ampoule, which was evacuated and filled with a nitrogen-bubbled monomer solution to immerse the fabric completely. The ampoule was placed in a temperature-controlled oven. Under these conditions, the radicals initiated graft polymerization. The monomer structures and grafting conditions are shown in Table 1. After graft polymerization, the fabric was washed with methanol to remove residual monomers and homopolymers and dried in an oven at 60 °C for more than 24 h. 

The degree of grafting and the density of alkyl chains of the grafted fabrics were calculated using the following equations.

Degree of grafting (%) = (W_g_ − W_0_)/W_0_ × 100 (1)

Density of alkyl chains (mmol/g) = 1000 × (W_g_ − W_0_)/M/W_g_ (2)

where W_0_ and W_g_ are the dry weights (mg) of the fabrics before and after graft polymerization, and M is the molecular weight of the monomers as shown in Table 1.

### 2.3. Loading of EHEP onto the Grafted Fabrics

A 50 wt.% EHEP solution of ethanol was uniformly dropped onto the grafted fabric for EHEP loading. The EHEP-loaded fabric was dried in a vacuum oven at 40 °C to remove the ethanol solvent. EHEP loading of the resulting fabric adsorbent was calculated by the following equation.

EHEP loading (mmol/g) = 1000 × (W_a_ − W_g_)/306/W_a_ (3)

where W_a_ is the dry weights (mg) of EHEP-loaded fabric, and 306 is the molecular weight of EHEP. The prepared fabric adsorbents with different monomers were named BMA-, HMA-, DMA-, and OMA-adsorbent, respectively. 

### 2.4. Characterization

Fourier transform infrared (FTIR) spectroscopic analysis was performed with an FTIR spectrophotometer in the attenuated total reflectance (ATR) mode (Spectrum One, PerkinElmer, Inc., Tokyo, Japan). The scanning range and resolution were 500–2500 cm^−1^ and 1 cm^−1^, respectively.

The hydrophobicity of the grafted fabric was examined by measuring the contact angle with a contact angle meter (CA-X, Kyowa Interface Science Co., Ltd., Tokyo, Japan).

### 2.5. Batch Adsorption Tests

The prepared fabric adsorbent was evaluated by batch adsorption tests. The test solution contained 100 ppm Dy (III) and 100 ppm Nd (III). The pH of the test solution was adjusted to 2.0 by ammonia water. The fabric adsorbent with a size of 2 cm × 2 cm was immersed in 50 mL of test solution in a glass bottle. The bottle was placed on a shaker and shaken at a rate of 150 rpm at 25 °C for 3.0 h. After the adsorption test, the adsorbent was washed with deionized water to remove the unadsorbed ions on them. 

To elute the adsorbed ions, the fabric adsorbent was immersed in 50 mL of 1.0 M HCl solution in a glass bottle, and the bottle was shaken at a rate of 150 rpm at 25 °C for 1.0 h. After elution, the fabric adsorbent was washed with deionized water and adsorption was repeated under the same conditions as the first adsorption test. 

The ion concentrations in the adsorption and elution solutions were analyzed before and after each test with an inductively coupled plasma optical emission spectrometer (ICP-OES, Optima 8300, PerkinElmer, Inc., Tokyo, Japan). The adsorptivity (mg/g) of the fabric adsorbent was calculated as follows.

Adsorptivity (mg/g) = 1000 × (C_0_ − C_i_) × V/W_a_ (4)

where C_0_ (mg/mL) and C_i_ (mg/mL) are the metal ion concentrations in the solution before and after the adsorption, respectively, and V (mL) is the volume of the solution.

### 2.6. Column Adsorption Tests 

For the column adsorption tests, the fabric adsorbent with a diameter of 7.0 mm was packed into a column with an inner diameter of 7.0 mm. The volume of the adsorbent packed in the column was 0.2 mL. The test solution (100 ppm Dy (III) and 100 ppm Nd (III), pH 2) was passed through the column at a space velocity (SV) of 100 h^−1^ at 25 °C. The SV is calculated by dividing the solution flow rate (mL/h) by the volume of adsorbent in the column (fixed at 0.2 mL in this study). A fraction collector was used to continuously collect the effluent from the column, and the ion concentrations were detected by ICP-OES. By plotting the relationship between C_i_ and bed volume (BV), the ion concentration curve of the effluent was obtained. Here, C_i_ is the ion concentration of the effluent at BV, and BV is calculated by dividing the total effluent volume from the column by the adsorbent volume (0.2 mL). 

The adsorptivity (mg/g) of the adsorbent packed in the column was calculated by the following equation

Adsorptivity (mg/g) = 1000 × ∑ (C_0_ − C_i_) ∆V_i_/W_a_ (5)

where ∆V_i_ (mL) and C_i_ (mg/mL) are the volume and concentration of each collected effluent during the adsorption, respectively. 

After the adsorption test, the adsorbent was thoroughly washed by passing deionized water through the column. Then, 1.0 M HCl solution of the eluent was passed through the column with a space velocity of 100 h^−1^ at 25 °C until no metal ions were detected in the effluent. The eluted amount (mg/g) and recovery ratio were calculated by the following equations.

Eluted amount (mg/g) = 1000 × ∑ C_i_ ∆V_i_/W_a_ (6)

Recovery ratio (%) = Eluted amount ⁄ Adsorptivity × 100(7)

where ∆V_i_ (mL) and C_i_ (mg/mL) are the volume and concentration of each collected effluent during the elution, respectively. 

After the elution test, the adsorbent in the column was thoroughly washed with deionized water and used for the adsorption test again to evaluate its stability. 

## 3. Results and Discussion

### 3.1. Synthesis of EHEP-Loaded Adsorbent

The adsorbent was prepared by the radiation-induced graft polymerization of methacrylate with a long alkyl chain onto PE/PP nonwoven fabric and the subsequent loading of EHEP by hydrophobic interaction and chain entanglement between the alkyl chains. Here, the EHEP organophosphorus compound has a special affinity for Dy (III) ions. The grafting results and the density of EHEP loading are summarized in Table 2. 

As shown in Table 2, four monomers with different alkyl chain lengths—BMA, HMA, DMA, and OMA—were radiation-grafted onto the fabrics. For comparison, the alkyl chain density in the grafted fabric was adjusted to be close to 2.0 mmol/g. For this reason, the degree of grafting was significantly different for each monomer and increased in proportion to the molecular weight of the grafted monomer. For example, to obtain a similar alkyl chain density of 2.0 mmol/g, the degree of grafting for the BMA is 51%, while it is 219% for the OMA. The latter is approximately four times higher than that of the former. 

To obtain similar alkyl chain densities of the grafted fabrics, BMA grafting was carried out by immersing the 10 kGy preirradiated fabric into a 5.0 wt.% BMA emulsion at 40 °C for 15 min, while for HMA grafting, a higher temperature of 60 °C and longer grafting time of 30 min were needed. We also carried out BMA grafting at 60 °C. However, the grafting rate was too fast to control the graft yielding. For monomers with longer alkyl chains, preirradiation doses higher than 100 kGy were used to generate more radicals in the fabrics. This is because the steric hindrance effects of the monomers inhibited the graft polymerization from reaching a high degree of grafting. Furthermore, a mixture solvent of methanol and water in the ratio of 1:1 was used for OMA grafting. Here, the addition of methanol to the monomer solution increased the affinity between the fabric and the monomer, thereby enhancing the radiation grafting [53]. 

The loading of EHEP onto the grafted fabric was achieved by dropping the EHEP solution of ethanol onto the grafted fabric to reach a loading density of approximately 1.2 mmol/g. After removing ethanol by evaporation, the adsorbent was obtained. 

### 3.2. Materials Characterization

**Figure 2:**
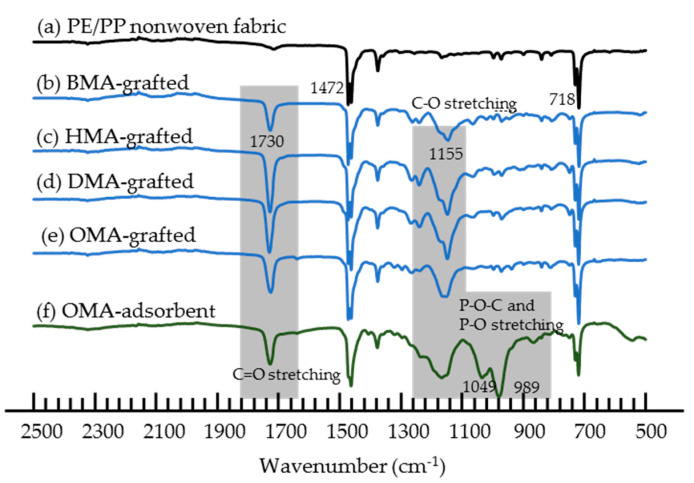
FTIR spectra of (**a**) PE/PP nonwoven fabric, (**b**) BMA-grafted PE/PP nonwoven fabric, (**c**) HMA-grafted PE/PP nonwoven fabric, (**d**) DMA-grafted PE/PP nonwoven fabric, (**e**) OMA-grafted PE/PP nonwoven fabric, and (**f**) OMA-adsorbent prepared by loading of EHEP onto the OMA-grafted PE/PP nonwoven fabric.

The FTIR results shown in Figure 2 confirmed that the BMA, HMA, DMA, and OMA monomers were graft polymerized onto the PE/PP nonwoven fabrics and EHEP was loaded onto the OMA-grafted fabric. The peaks of the PE/PP nonwoven fabric only appeared at 1472, 1462, 1375, 731, and 718 cm^−1^, corresponding to the characteristic absorptions of PE [54], indicating that the PP fiber was completely coated by PE. After grafting, new peaks at 1730 and 1155 cm^−1^, attributed to the C=O and C–O stretching of methacrylate, respectively, were observed (Figure 2b–e) [55,56]. After loading EHEP onto the OMA-grafted fabric, new peaks at 1250 (P–O–C), 1050 (P–O–C), and 980 (P=O) cm^−1^ were observed, as shown in Figure 2f [25]. These results indicated that the methacrylate monomers were grafted onto the fabrics and EHEP was loaded onto the OMA-grafted fabric. 

**Figure 3:**
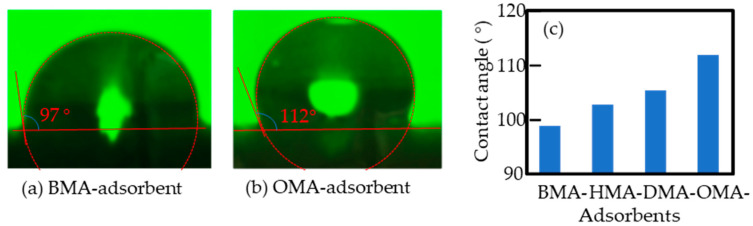
Water droplets on the BMA-adsorbent (**a**) and OMA-adsorbent (**b**). Contact angle of the water droplets on the four fabric adsorbents (**c**).

The surface properties of the BMA-, HMA-, DMA-, and OMA-grafted fabrics were evaluated by a contact angle meter. A high contact angle indicates the high hydrophobicity of the sample. Pictures of water droplets on the surface with the smallest and largest contact angles are shown in Figure 3a, b, respectively. The contact angle of the BMA-grafted fabric was 97° (Figure 3a), while that of the OMA-grafted fabric was 112° (Figure 3b). For comparison, the contact angles of the grafted fabrics are summarized in Figure 3c. The contact angle increased with the increase of the alkyl chain length of the grafted monomers. The OMA-grafted fabric had the highest hydrophobicity due to the longest alkyl chains of the grafted monomers as well as the highest degree of grafting (see Table 2). It was expected that the grafted fabric with high hydrophobicity was more conducive to the physical bonding of the alkyl chain of EAEH for loading.

### 3.3. Batch Adsorption Tests

The adsorbent performance was evaluated in advance by a batch adsorption test. The aqueous solution of 100 ppm Dy (III) and 100 ppm Nd (III) at pH 2 was used as the adsorption solution. After adsorption, the adsorbent was immersed in 1.0 M HCl solution to completely elute the adsorbed ions and washed with adequate water to conduct the adsorption test again. 

**Figure 4:**
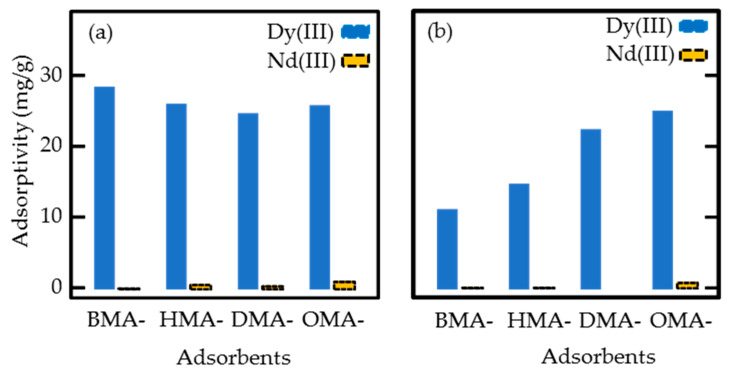
The Dy (III) and Nd (III) adsorptivity of the four nonwoven fabric adsorbents. (**a**) First adsorption test. (**b**) Second adsorption test using the refreshed adsorbents after elution and washing. Initial adsorption solution: 100 ppm Dy (III) and 100 ppm Nd (III) at a pH of 2.0 at 25 °C.

The results of the batch adsorption test are shown in Table 3 and Figure 4. In the first adsorption test, all adsorbents had similar Dy (III) adsorptivity around 25.0 mg/g. The EHEP loaded in the fabric is a cationic extractant, which is known to extract metal ions from aqueous solution and can be labeled HA. The adsorption is an ion-exchange process in which one Dy (III) ion combines three EHEPs to form a DyA_3_ structure in the adsorbent [57,58]. The similar adsorptivity was due to the similar EHEP loading (1.20 mmol/g) of the four adsorbents. However, the Nd (III) adsorptivity for each adsorbent was considerably small (less than 1.0 mg/g). Therefore, the EHEP-loaded adsorbents had a high adsorption selectivity for Dy (III) and could be used for separation and recovery. 

In the repeated adsorption tests, the OMA-adsorbent retained a high Dy (III) adsorptivity of 25.3 mg/g. In contrast, the Dy (III) adsorptivities of BMA-, HMA-, and DMA-adsorbents were significantly reduced to were 11.4, 15.0, and 22.7 mg/g, respectively. The decrease of Dy (III) adsorptivity might be due to the loss of EHEP loaded in the fabric during the repeated tests. As shown in Table 3, after repeated adsorption tests, the weight of the OMA-adsorbent was almost unchanged, while the weight of the BMA-adsorbent was reduced by 26%. The shorter the alkyl chain length of the grafted monomer, the more the weight of the adsorbent decreased due to the loss of EHEP. According to these results, the OMA-adsorbent with the longest alkyl chain was chosen for the column adsorption test.

### 3.4. Column Adsorption Tests

**Figure 5:**
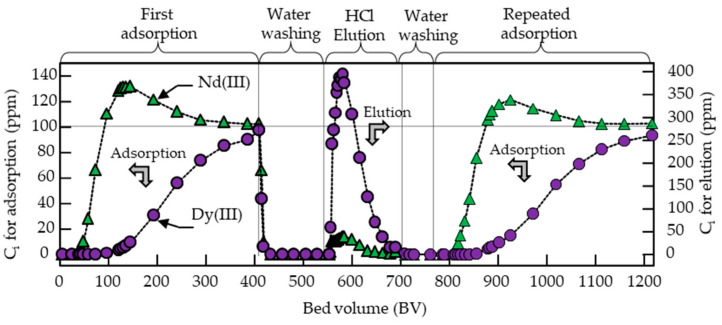
Profile of the adsorption and elution of Dy (III) and Nd (III) ions with an OMA-adsorbent. Adsorption solution: 100 ppm Dy (III) and 100 ppm Nd (III), pH 2.0; elution solution: 1.0 M HCl; space velocity (SV) = 100 h^−1^; temperature = 25 °C; the total 1200 BV means that the adsorption–elution–adsorption process was operated for 12 h under the fixed space velocity of 100 h^−1^.

Column adsorption and elution were carried out using the same adsorption and elution solutions as the above batch tests. The solution was passed through the column at a space velocity of 100 h^−1^. As shown in Figure 5, the Dy (III) was completely adsorbed up to a higher bed volume (BV) of 80. After that, the concentration of Dy (III) in the effluent gradually increased, reaching 98 ppm at a BV of 400 (similar to the concentration of the fed solution, 100 ppm). The total Dy (III) adsorbed from the solution was calculated using Equation (5) to be 43.6 mg/g. The adsorption is an ion-exchange process between the metal ions and the proton of EHEP loaded in the fabrics; that is, one Dy (III) ion can bond with three phosphate groups. Therefore, for a 1.2 mmol/g EHEP-loaded adsorbent, the calculated adsorption capacity is close to 64.8 mg/g. The detected value of 43.6 mg/g is lower than the calculated value, which is due to the adsorption equilibria at the low Dy (III) concentration of the feed solution. Even then, it is still much higher than in the case of using hybrid silica nanoparticles, as reported by Topel et al., where the Dy (III) adsorption is 0.019 mmol/g or 30.9 mg/g [57]. In contrast, the Nd (III) was completely adsorbed up to a lower BV of 40, and the Nd (III) concentration rapidly increased up to 130 ppm at a BV of 144, which was higher than that of the fed solution (100 ppm). This is because the adsorbed Nd (III) was replaced by Dy (III), indicating that the OMA-adsorbent was favorable for Dy (III) adsorption. The Nd (III) adsorptivity of the adsorbents in the column was also calculated using Equation (5) to be 4.2 mg/g, which was one-tenth of the Dy (III) adsorption. 

The adsorbed Dy (III) and Nd (III) were eluted by passing 1.0 M HCl solution through the column. The maximum concentrations of Dy (III) and Nd (III) in the effluent were 373 and 38 ppm, respectively. The recovery ratios of Dy (III) and Nd (III) calculated using Equations (6) and (7) were 99% and 98%, respectively, indicating that almost all metal ions were eluted by the 1.0 M HCl solution within a BV of 160 (from 550 to 710 BV in Figure 5). 

After the first adsorption, water washing, HCl elution, and water washing, the repeated column test was continued (Figure 5). The concentration curves of Dy (III) and Nd (III) for the repeated adsorption test show similar behavior as the first adsorption test. These results indicate that the OMA-adsorbent was stable for repeated use in the separation and recovery of Dy (III) and Nd (III) ions from an aqueous solution.

## 4. Conclusions

A fabric adsorbent for the separation and recovery of Dy (III) and Nd (III) from an aqueous solution was successfully prepared by graft polymerization of methacrylate with a long alkyl chain onto the nonwoven fabric and loading EHEP by hydrophobic interaction and chain entanglement between the alkyl chains. 

In the batch adsorption tests, the adsorbents showed a high Dy (III) adsorptivity above 25.0 mg/g and a low Nd (III) adsorptivity below 1.0 mg/g, indicating that the adsorbents had a high Dy (III) selective adsorption. However, only the OMA-adsorbent with the longest alkyl chain was stable and retained its high Dy (III) adsorption performance in repeated adsorption tests. 

In the column adsorption test with the OMA-adsorbent, the adsorptivities of Dy (III) and Nd (III) were 43.6 and 4.2 mg/g, respectively. The Dy (III) adsorptivity was approximately ten times higher than that of the Nd (III) adsorptivity. Similar adsorption performance of the adsorbents was observed in the repeated tests. These results demonstrate that the OMA-adsorbent was stable for repeated use. The high stability of the OMA-adsorbents due to the loss of EHEP was suppressed by the strong hydrophobic interaction and chain entanglement between the long alkyl chains. 

The OMA-adsorbent can be synthesized easily and economically by immersing the irradiated nonwoven fabric in the monomer solution and EHEP solution in sequence. The obtained adsorbent can be used in batch mode or column mode without any other separation process. Even if the adsorbent is operated in a strong acid, it is stable without any weight loss. Furthermore, the adsorbent has a high selectivity to Dy (III) ions. Therefore, the OMA-adsorbent developed in this study can effectively separate and recover Dy (III) and Nd (III) from an aqueous solution and is expected to contribute to the recovery of rare-earth metals from NdFeB permanent magnet scraps in the future.

## Figures and Tables

**Table 1:** Methacrylate monomers and grafting conditions used in this study.

			Grafting Conditions **		
Name	Molecular Structures	M *	Dose *** (kGy)	Temp. (°C)	Time (min)
Butyl methacrylate (BMA)	CH_2_=CCH_3_COOC_4_H_9_	142	10	40	15
Hexyl methacrylate (HMA)	CH_2_=CCH_3_COOC_6_H_13_	170	10	60	30
Dodecyl methacrylate (DMA)	CH_2_=CCH_3_COOC_12_H_25_	254	300	60	180
Octadecyl methacrylate (OMA)	CH_2_=CCH_3_COOC_18_H_37_	338	100	60	120

* M is the molecular weight of the monomers; ** For the monomer solutions, monomer concentrations were fixed at 5.0 wt.% in water for BMA, HMA, and DMA, and in a water/methanol mixture solvent (1:1 in weight) for OMA; 0.5 wt.% of Tween 20 surfactant was added to the monomer solutions. *** Preirradiation was performed at −80 °C (dry ice) in an oxygen-free environment.

**Table 2:** Degree of grafting and alkyl group density of the grafted fabrics, and the EHEP loading of the corresponding adsorbents.

Grafted Monomers	Degree of Grafting (%)	Alkyl Group Density * (mmol/g)	EHEP Loading ** (mmol/g)
Butyl methacrylate (BMA)	51	2.39	1.24
Hexyl methacrylate (HMA)	62	2.24	1.26
Dodecyl methacrylate (DMA)	102	1.99	1.24
Octadecyl methacrylate (OMA)	219	2.03	1.22

* Alkyl group density of the monomer-grafted fabric was calculated using Equation (2); ** EHEP loading was calculated by the weight increase of the grafted fabric before and after EHEP loading using Equation (3).

**Table 3:** Summary of the first and repeated batch adsorption tests.

Adsorbents	1st Adsorption *			Repeated Adsorption **			W_a_/W_b_
	W_b_ (mg)	C_Dy-1_ (mg/g)	C_Nd-1_ (mg/g)	C_Dy-r_ (mg/g)	C_Nd-r_ (mg/g)	W_a_ (mg)	
BMA	54	28.6	0.1	11.4	0.1	40	0.74
HMA	66	26.2	0.6	15.0	0.1	51	0.77
DMA	80	24.9	0.4	22.7	0	74	0.93
OMA	86	26.0	1.0	25.3	0.8	82	0.95

* First adsorption was performed using the new adsorbent, and W_b_ is the dry weight of the new adsorbent, C_Dy-1_ and C_Nd-1_ are the Dy(III) and Nd(III) adsorptivities of the first adsorption, respectively; ** Repeated adsorption was performed after the adsorbent diluted and adequate water-washed, C_Dy-r_ and C_Nd-r_ are the Dy(III) and Nd(III) adsorptivities of the repeated adsorption, respectively, and W_a_ is the dry weight of the used adsorbent after the repeated adsorption and dilution.
